# Determinants of decision-making in orthognathic surgery among patients in Ha’il region, Saudi Arabia

**DOI:** 10.1080/07853890.2025.2477305

**Published:** 2025-10-08

**Authors:** Abdullah F. Alshammari, Yousef E. Alenezi, Lamia H. Alharbi, Ghadah D. Aldakhayel, Ahmed A. Madfa, Khlood A. Alkurdi

**Affiliations:** ^a^ Department of Basic Dental and Medical Science, College of Dentistry, University of Ha’il, Ha’il, Kingdom of Saudi Arabia; ^b^ Medical and Diagnostic Research Center, University of Hail, Hail, Saudi Arabia; ^c^ Dental Intern, College of Dentistry, University of Ha’il, Ha’il, Kingdom of Saudi Arabia; ^d^ Department of Restorative Dental Science, College of Dentistry, University of Ha’il, Ha’il, Kingdom of Saudi Arabia; ^e^ Ministry of Health, Qassim Health Cluster, King Saud Hospital, Unayzah, Kingdom of Saudi Arabia; ^f^ Institute of Dentistry, Queen Mary University of London, London, United Kingdom

**Keywords:** Orthognathic surgery, decision-making, dentofacial deformities, aesthetic concern, functional concerns, healthcare professionals, social stigma, cultural beliefs, Saudi Vision 2030

## Abstract

**Introduction:**

Orthognathic surgery is essential for addressing severe dentofacial deformities, improving both function and aesthetics. In Ha’il, Saudi Arabia, the decision-making process for this surgery is influenced by a range of medical, psychological, and social factors. This study examined the key factors influencing patients’ decisions to undergo orthognathic surgery in Ha’il.

**Methods:**

A cross-sectional study was conducted from 2019–2023, involving 27 patients in Ha’il who either required or had undergone orthognathic surgery. Data were collected through structured interviews covering demographics, medical history, consultation experiences, and decision influences. Statistical analyses, including chi-square tests and logistic regression models, were performed with a significance level of *p* < 0.05.

**Results:**

Among the participants, 59.3% were male, and Class III malocclusion was the most prevalent deformity (59.3%). Aesthetic concerns were the primary motivator for 48.1% of patients, while functional concerns drove 18.5%. Only 44.4% of participants reported receiving comprehensive information about the surgery’s risks and benefits. Healthcare professionals were the main decision influencers, cited by 51.9% of participants, while social stigma impacted 25.9%. Cultural beliefs had minimal influence on the decision-making process.

**Conclusion:**

The decision to undergo orthognathic surgery in Ha’il is predominantly driven by aesthetic concerns, with healthcare professionals playing a crucial role in patient education. Cultural beliefs have little influence, though social stigma is a notable factor. Enhancing patient education could improve decision-making, aligning with Saudi Vision 2030’s healthcare goals.

## Introduction

Orthognathic surgery, also known as corrective jaw surgery, is a critical intervention for individuals with severe dentofacial deformities that cannot be corrected through orthodontic treatment alone. It involves the precise repositioning of the jaw to improve functional aspects, such as chewing, speaking, and breathing, and aesthetic concerns that can significantly impact an individual’s self-confidence and social interactions [1]. An individual’s decision to undergo orthognathic surgery is often complex and multifaceted, involving considerations that extend beyond the immediate clinical indications to encompass personal, psychological, and social factors [2].

Numerous factors influence the decision-making process for people contemplating orthognathic surgery. These include the degree of facial asymmetry or malocclusion, how the issue affects their day-to-day activities, and their expectations for the results of surgery [2]. Eating, speaking, and breathing problems are among the functional impairments linked to these diseases, and they frequently act as the main drivers behind reconstructive surgery. Nonetheless, a person’s wish to enhance their facial appearance and, consequently, their sense of self-worth and social confidence, has a big impact on their decision to have surgery [3].

The prevalence of orthognathic surgery varies significantly across regions, reflecting disparities in healthcare access, cultural attitudes towards facial aesthetics, and the availability of specialised surgical expertise. In many Western countries, such as the United States and parts of Europe, orthognathic surgery is relatively common, supported by advanced healthcare systems that have the expertise and financial resources required for this procedure. For example, in Sweden, orthognathic surgery is performed on approximately 6.3 per 100,000 individuals annually [4]. In contrast, its prevalence in developing regions, such as parts of Asia and Africa, is lower, often due to limited access to specialised care and financial barriers. However, in countries like South Korea, where the cultural emphasis on facial aesthetics is strong, orthognathic surgery is more prevalent, with patients often undergoing the procedure for both functional and aesthetic reasons [5].

In Saudi Arabia, the prevalence of orthognathic surgery is increasing, driven by advancements in healthcare and a growing recognition of the importance of facial aesthetics. However, the specific prevalence and factors influencing the decision-making process among potential surgery candidates in the Ha’il region remain underexplored [6]. This highlights a significant gap in the understanding of local healthcare needs and the factors that guide patients in their decision-making processes regarding this complex and often life-altering surgery.

The decision-making process for patients considering orthognathic surgery is particularly complex in regions like Ha’il, where patients’ awareness and understanding of the procedure may be limited. Despite the importance of orthognathic surgery for improving quality of life, there is a lack of comprehensive data on the prevalence of conditions that necessitate this surgery in Ha’il, as well as on the factors that influence patients’ decisions. The Saudi Vision 2030 highlights Saudi Arabia’s goals regarding the advancement of its healthcare services and enhancing the quality of life for all citizens. It also underscores the need to improve the support offered to patients during the decision-making process. However, the lack of clear data and public awareness about orthognathic surgery in Ha’il poses a challenge to achieving these objectives [7].

In the Ha’il region, the decision to undergo orthognathic surgery is influenced by a variety of factors, including the availability of surgery-related information, the perceived risks and benefits, and the social and cultural attitudes towards facial aesthetics and surgical interventions [8]. Patients may also be influenced by their interactions with healthcare providers, who play a crucial role in guiding them through the decision-making process. In many cases, patients may not have sufficient information or support to make fully informed decisions, leading to uncertainty or reluctance to proceed with the surgery. Therefore, this study aimed to investigate the factors that influence the decision-making process among patients indicated for orthognathic surgery in Ha’il, Saudi Arabia.

## Materials and methods

### Study design and settings

This study used an interview-based cross-sectional study design to explore the factors influencing the decision-making process among patients indicated for orthognathic surgery in Ha’il, Saudi Arabia. The participants for the study were recruited from Maxillofacial Department at Leading Hospital in Ha’il City, where individuals diagnosed with malocclusions who either required or had undergone orthognathic surgery were selected for study inclusion. The data collection period spanned from 2019–2023, enabling a comprehensive examination of patient experiences and decision-making processes over time.

### Data collection procedure and outcome measures

Participants who were aged 18 or older, lived in Ha’il, were indicated for or had undergone orthognathic surgery, and who consented to participate and are otherwise healthy were included in the study. Those who did not meet these criteria were excluded. Additionally, Individuals with cognitive impairments, such as dementia, intellectual disabilities or neoplastic malformations, that might hinder their assessment were excluded. Patients with incomplete medical records relevant to orthognathic surgery were excluded as well.

Data were gathered through an interviewer-administered questionnaire. The questionnaire was structured into eight sections: demographic information, medical history, consultation and treatment details, decision influencers, emotional and post-surgery experiences, knowledge and awareness, social and cultural influences, and post-surgery reflections (see appendix 1). To ensure data validity, the principal investigator re-contacted 25% of the participants (7 out of 27) one month after the initial data collection. The investigator re-administered the questionnaire to these participants, and the responses were compared to the initial data to verify consistency.

### Ethical considerations

An application for ethical permission was submitted to the Institutional Review Board of the Research and Studies Department at the University of Ha’il (H-2024-405). Ethical approval was granted. Verbal informed consent was obtained from each participant after they were informed about the purpose and methods of this research.

### Statistical analysis

The data obtained were organized and analysed using the Statistical Package for Social Sciences (SPSS) software version 23 (SPSS Inc., Chicago, IL, USA). Descriptive statistics were used to summarise the frequencies of different decision-making factors and the demographic features of the study participants. Chi-square test was applied to explore the relationships between the decisions made and the underlying reasons. A p-value of less than 0.05 was considered statistically significant.

## Results

Fifty-seven electronic medical records from patients who were indicated for or had undergone orthognathic surgery were retrieved. Thirty cases were excluded for not meeting the inclusion criteria (e.g. the presence of cleft lip and/or palate, incorrect or missing contact information, or non-response/refusal to participate). Therefore, 27 cases were included in the final analysis.

Of the 27 included participants, 59.3% were male and 40.7% were female. The most common type of expressed deformity was Class III (59.3%), followed by Class II (22.2%) and Class I (18.5%). Only 14.8% of participants had a family history of these deformities. Regarding participants’ knowledge about their medical condition, 44.4% reported receiving comprehensive information about the risks and benefits of the surgery, while 25.9% received incomplete information, and 29.7% received no information. Information on pre- and post-surgical preparations was similarly distributed, with 40.7% indicated that they had received the information, while an equal percentage of 40.7% stated that they had not. Additionally, 18.5% reported that they were not fully informed (Table 1). Among those who received information, the primary information source was doctors (51.9%), followed by the internet (29.6%) and social media (7.4%). A small proportion of participants (3.7%) relied on friends and family for information.

**Table 1.** Patient demographics and influences on orthognathic surgery decisions.

Variables		Frequency	Percent
Gender	Male	16	59.3%
	Female	11	40.7%
Age	18-23	11	40.7%
	24-29	10	37.0%
	30-44	6	22.2%
Level of education	High school	10	37.0%
	Diploma	2	7.4%
	University level	15	55.6%
Presence of systemic disease	Yes	4	14.8%
	No	23	85.2%
Type of deformity	Class I	5	18.5%
	Class II	6	22.2%
	Class III	16	59.3%
Patient’s main concern	Aesthetic	13	48.1%
	Function	5	18.5%
	Both	6	22.2%
	Others	3	11.1%
Presence of related family history	Yes	4	14.8%
	No	23	85.2%
Whether patients received information about the risks and benefits of the surgery	Yes	12	44.4%
	No	8	29.6%
	Not fully	7	25.9%
Whether patients received information on pre- and post-surgical preparation	Yes	11	40.7%
	No	11	40.7%
	Not fully	5	18.5%
Sources used by patients to learn about orthognathic surgery	Doctor	14	51.9%
	Internet	8	29.6%
	Social media	2	7.4%
	Friends/family	1	3.7%
	Other	2	7.4%
Effect of social media on patient decisions	Effect	8	29.6%
	Little effect	8	29.6%
	did not affect	11	40.7%
Effect of family opinion on patient decision	Effect	5	18.5%
	Little effect	6	22.2%
	did not affect	16	59.3%
The extend to which patients’ expected outcomes affected their decision	not important	1	3.7%
	Slightly important	1	3.7
	Very important	9	33.3
	It was the main factor	16	59.3
Patients’ major concerns about doing the surgery.	Possible pain	8	29.6
	Outcome	7	25.9
	Nothing	9	33.3
	Others	3	11.1
Patients’ emotional expression prior surgery	Very anxious	4	14.8
	Slightly anxious	2	7.4
	Natural	14	51.9
	Excited	2	7.4
	N\A	5	18.5
Communication with the assigned Healthcare Team	Excellent	14	51.9
	Good	5	18.5
	Average	7	25.9
	Poor	1	3.7
Awareness of non-surgical alternatives to orthognathic surgery	Yes	17	63.0
	No	10	37.0
If yes, where the options were considered	Yes	14	51.9
	No	3	11.1
	N\A	10	37.0
Accuracy level of the information given to patients regarding their surgery	Very accurate	13	48.1
	Somewhat	11	40.7
	Not accurate	3	11.1
The influence of cultural beliefs and norms on patients’ surgery decision	Not at all	18	66.7
	Little	5	18.5
	A lot	3	11.1
	It was the main factor	1	3.7
Any experience social stigma or negative reactions from others regarding your decision to undergo surgery	Yes	7	25.9
	No	20	74.1
Whether patients recommend orthognathic surgery to others after they received the treatment	Yes	12	44.4
	Maybe	15	55.6

Aesthetic concerns were a significant issue for 48.1% of participants, while only 18.5% prioritized their medical needs (Table 1). Anxiety was reported by 14.8% of participants. Communication with the healthcare team was rated as excellent by 51.9% of participants, good by 18.5%, average by 25.9%, and poor by 3.7%. Cultural beliefs and norms were not a factor for 66.7% of participants; however, 3.7% considered them to be the main factor influencing their decision (Table 1). Social stigma or negative reactions regarding the decision to undergo surgery were experienced by 25.9% of participants. After receiving treatment, 44.4% would recommend orthognathic surgery to others, while 55.6% remained unsure.

### Impact of gender on orthognathic surgery decisions

The analysed data revealed that males were more likely to report systemic diseases compared to females, and a higher percentage of males had Class III deformities compared to females (Table 2). Aesthetic concerns were equally important for both genders. However, functional concerns were more prominent among males (25.0%) than females (9.1%), while females were more likely to be concerned with both aesthetics and function (36.4%).

**Table 2.** Analysis of decision-making factors in relation to gender.

Orthognathic Decision		Gender	
		Male	Female
Presence of systemic disease	Yes	18.8%	9.1%
	No	81.3%	90.9%
Type of deformity	Class I	12.5%	27.3%
	Class II	18.8%	27.3%
	Class III	68.8%	45.5%
Patient’s main concern	Aesthetic	50.0%	45.5%
	Function	25.0%	9.1%
	Both	12.5%	36.4%
	Others	12.5%	9.1%
Presence of related family history	Yes	18.8%	9.1%
	No	81.3%	90.9%
Whether patients received information about the risks and benefits of the surgery	Yes	56.3%	27.3%
	No	31.3%	27.3%
	Not fully	12.5%	45.5%*
Whether patients received information on pre- and post-surgical preparation	Yes	50.0%	27.3%
	No	37.5%	45.5%
	Not fully	12.5%	27.3%
Sources used by patients to learn about orthognathic surgery	Doctor	56.3%	45.5%
	Internet	18.8%	45.5%
	Social media	12.5%	0.0%
	Friends/family	0.0%	9.1%
	Other	12.5%	0.0%
Effect of social media on patient decisions	Effect	18.8%	45.5%
	Little effect	31.3%	27.3%
	Did not affect	50.0%	27.3%
Effect of family opinion on patient decision	Effect	25.0%	9.1%
	Little effect	25.0%	18.2%
	Did not affect	50.0%	72.7%
The extend to which patients’ expected outcomes affected their decision	Not important	6.3%	0.0%
	Slightly important	6.3%	0.0%
	Very important	43.8%	18.2%
	It was the main factor	43.8%	81.8%*
Patients’ major concerns about doing the surgery	Possible pain	43.8%	9.1%
	Outcome	18.8%	36.4%
	Nothing	25.0%	45.5%
	Others	12.5%	9.1%
Patients’ emotional expression prior surgery	Very anxious	18.8%	9.1%
	Slightly anxious	0.0%	18.2%
	Natural	37.5%	72.7%*
	Excited	12.5%	0.0%
	N\A	31.3%	0.0%
Communication with the assigned Healthcare Team	Excellent	68.8%	27.3%
	Good	12.5%	27.3%
	Average	18.8%	36.4%*
	Poor	0.0%	9.1%
Awareness of non-surgical alternatives to orthognathic surgery	Yes	62.5%	63.6%
	No	37.5%	36.4%
If yes, where the options were considered	Yes	56.3%	45.5%
	No	6.3%	18.2%
	N\A	37.5%	36.4%
Accuracy level of the information given to patients regarding their surgery	Very accurate	56.3%	36.4%
	Somewhat	31.3%	54.5%
	Not accurate	12.5%	9.1%
The influence of cultural beliefs and norms on patients’ surgery decision	Not at all	50.0%	90.9%
	Little	31.3%	0.0%
	A lot	18.8%	0.0%
	It was the main factor	0.0%	9.1%
Any experience social stigma or negative reactions from others regarding your decision to undergo surgery	Yes	37.5%	9.1%
	No	62.5%	90.9%
Whether patients recommend orthognathic surgery to others after they received the treatment	Yes	56.3%	27.3%
	Maybe	43.8%	72.7%

*P-value < 0.05.

Males were more likely to have a related family history (18.8%) compared to females (9.1%). Information about the risks and benefits of surgery was more thoroughly received by males, with a significant proportion of females reporting that they received incomplete information. In terms of information sources, males relied more on doctors (56.3%), whereas females more frequently used the internet (45.5%). Social media had a greater impact on the decision-making process for females (45.5%) than males (18.8%). Family opinions played a more significant role for males, with 25.0% reporting it as influential compared to 9.1% of females.

Expected outcomes were the main factor for 81.8% of females in their decision-making process, compared to 43.8% of males. Males were more concerned about possible pain compared to females, who were more likely to report no concerns. Prior to surgery, more females reported feeling feeling both physically and mentally well. (72.7%) compared to males (37.5%), with a notable proportion of males feeling very anxious (18.8%) or opting not to disclose their emotions (31.3%). Communication with the healthcare team was rated as excellent by 68.8% of males compared to 27.3% of females, who more frequently rated it as average or poor (Table 2).

Awareness of non-surgical alternatives was similar between genders; however, a higher percentage of males actively considered these alternatives compared to females. Cultural beliefs had no influence on the surgical decision-making process for 90.9% of females, whereas only 50.0% of males reported no influence, while 18.8% indicated it had a significant impact. Social stigma was reported to be influential by 37.5% of males compared to 9.1% of females. Post-surgery, 56.3% of males stated they would recommend orthognathic surgery to others, while 72.7% of females were uncertain. The Statistical association between gender and various variables were recorded in Table 2.

### Impact of age on orthognathic surgery decisions

Table 3 outlines the frequency distribution of various variables according to participant age. Class III deformities were most prevalent in the 24–29 age group (70.0%), while Class I deformities were more frequent in the 30–44 age group (33.3%). Aesthetic concerns were the primary motivation for most participants aged 24–29 (60.0%), whereas functional concerns were most prominent in the 18–23 age group (36.4%). The oldest age group (30–44) showed a balanced concern for both aesthetics and function (50.0% for each category).

**Table 3.** Analysis of decision-making factors in relation to age.

Orthognathic Decision		Age Group		
		18–23	24–29	30–44
Presence of systemic disease	Yes	9.1%	20.0%	16.7%
	No	90.9%	80.0%	83.3%
Type of deformity	Class I	18.2%	10.0%	33.3%
	Class II	27.3%	20.0%	16.7%
	Class III	54.5%	70.0%	50.0%
Patient’s main concern	Aesthetic	36.4%	60.0%	50.0%
	Function	36.4%	10.0%	0.0%
	Both	18.2%	10.0%	50.0%
	Others	9.1%	20.0%	0.0%
Presence of related family history	Yes	18.2%	10.0%	16.7%
	No	81.8%	90.0%	83.3%
Whether patients received information about the risks and benefits of the surgery	Yes	36.4%	60.0%	33.3%
	No	36.4%	20.0%	33.3%
	Not fully	27.3%	20.0%	33.3%
Whether patients received information on pre- and post-surgical preparation	Yes	27.3%	60.0%	33.3%
	No	54.5%	30.0%	33.3%
	Not fully	18.2%	10.0%	33.3%
Sources used by patients to learn about orthognathic surgery	Doctor	72.7%	60.0%	0.0%
	Internet	0.0%	30.0%	83.3%
	Social media	9.1%	10.0%	0.0%
	Friends/family	0.0%	0.0%	16.7%
	Other	18.2%	0.0%	0.0%
Effect of social media on patient decisions	Effect	18.2%	40.0%	33.3%
	Little effect	36.4%	30.0%	16.7%
	Did not affect	45.5%	30.0%	50.0%
Effect of family opinion on patient decision	Effect	9.1%	20.0%	33.3%
	Little effect	36.4%	10.0%	16.7%
	Did not affect	54.5%	70.0%	50.0%
The extend to which patients’ expected outcomes affected their decision	Not important	9.1%	0.0%	0.0%
	Slightly important	9.1%	0.0%	0.0%
	Very important	27.3%	40.0%	33.3%
	It was the main factor	54.5%	60.0%	66.7%
Patients’ major concerns about doing the surgery	Possible pain	27.3%	20.0%	50.0%*
	Outcome	9.1%	50.0%	16.7%
	Nothing	45.5%	20.0%	33.3%
	Others	18.2%	10.0%	0.0%
Patients’ emotional expression prior surgery	Very anxious	0.0%	30.0%	16.7%
	Slightly anxious	0.0%	10.0%	16.7%
	Natural	63.6%*	40.0%	50.0%
	Excited	9.1%	10.0%	0.0%
	N\A	27.3%	10.0%	16.7%
Communication with the assigned Healthcare Team	Excellent	27.3%	70.0%*	66.7%
	Good	36.4%	0.0%	16.7%
	Average	36.4%	20.0%	16.7%
	Poor	0.0%	10.0%	0.0%
Awareness of non-surgical alternatives to orthognathic surgery	Yes	54.5%	60.0%	83.3%
	No	45.5%	40.0%	16.7%
If yes, where the options were considered	Yes	54.5%	40.0%	66.7%
	No	0.0%	20.0%	16.7%
	N\A	45.5%	40.0%	16.7%
Accuracy level of the information given to patients regarding their surgery	Very accurate	36.4%	70.0%	33.3%
	Somewhat	45.5%	20.0%	66.7%
	Not accurate	18.2%	10.0%	0.0%
The influence of cultural beliefs and norms on patients’ surgery decision	Not at all	72.7%	50.0%	83.3%
	Little	27.3%	20.0%	0.0%
	A lot	0.0%	30.0%	0.0%
	It was the main factor	0.0%	0.0%	16.7%
Any experience social stigma or negative reactions from others regarding your decision to undergo surgery	Yes	18.2%	50.0%	0.0%
	No	81.8%	50.0%	100.0%
Whether patients recommend orthognathic surgery to others after they received the treatment	Yes	36.4%	70.0%	16.7%
	Maybe	63.6%	30.0%	83.3%

*P-value < 0.05.

Regarding family history, most participants, regardless of age group, reported no related family history, with the 24–29 age group showing the highest percentage of no family history (90.0%). Information about the risks and benefits of surgery was most thoroughly received by patients in the 24–29 age group (60.0%), while those aged 18–23 and 3–44 reported mixed experiences, with 36.4% and 33.3% receiving incomplete information, respectively.

In terms of pre- and post-surgical preparation, the 24–29 age group had the highest proportion of participants who stated they received information (60.0%), while the 18–23 age group had the highest percentage of patients who did not receive any preparation information (54.5%). The primary source of information for the 18–23 age group was doctors (72.7%), while the 30–44 age group relied more on the Internet (83.3%). Social media influenced the decisions of 40.0% of the 24–29 age group, while family opinions were more influential for the 30–44 age group (33.3%).

Awareness of non-surgical alternatives was highest among the 30–44 age group (83.3%), with most participants in this group also considering these options. The accuracy of information provided was rated very accurate by 70.0% of the 24–29 age group, while most of the 30–44 age group rated it as somewhat accurate (66.7%). Cultural beliefs had no influence on the decision-making process for most of the 30–44 age group (83.3%), while 30.0% of the 24-29 age group reported some influence.

Social stigma was most frequently reported by the 24–29 age group (50.0%), while the 30–44 age group reported no social stigma or negative reactions. After receiving treatment, 70.0% of the 24–29 age group stated that they would recommend orthognathic surgery to others, compared to 36.4% of the 18–23 age group. Most of the 30–44 age group remained uncertain (83.3%). The statistical relationship between different age groups and various variables was noted in Table 3.

### Impact of level of education on orthognathic surgery decisions

Table 4 details the frequency distribution of various variables according to participants’ levels of education. Aesthetic concerns were a significant factor across all educational levels, with 50.0% of diploma holders, 46.7% of university-educated participants, and 38.5% of high school graduates citing it as their main concern. Functional concerns were most prominent among high school graduates (60.0%), while diploma holders reported no concerns.

**Table 4.** Analysis of decision-making factors in relation to education.

Orthognathic Decision		Level of Education		
		High school	Diploma	University level
Presence of systemic disease	Yes	0.0%	50.0%	20.0%
	No	100.0%*	50.0%	80.0%
Type of deformity	Class I	40.0%	0.0%	20.0%
	Class II	16.7%	0.0%	33.3%
	Class III	43.8%	100.0%	46.7%
Patient’s main concern	Aesthetic	38.5%	50.0%	46.7%
	Function	60.0%	0.0%	13.3%
	Both	16.7%	50.0%	26.7%
	Others	10.0%	0.0%	13.3%
Presence of related family history	Yes	10.0%	0.0%	20.0%
	No	90.0%	100.0%	80.0%
Whether patients received information about the risks and benefits of the surgery	Yes	40.0%	100.0%*	40.0%
	No	30.0%	0.0%	33.3%
	Not fully	30.0%	0.0%	26.7%
Whether patients received information on pre- and post-surgical preparation	Yes	30.0%	100.0%	40.0%
	No	40.0%	0.0%	46.7%
	Not fully	30.0%	0.0%	13.3%
Sources used by patients to learn about orthognathic surgery	Doctor	50.0%	50.0%	53.3%
	Internet	20.0%	50.0%	33.3%
	Social media	10.0%	0.0%	6.7%
	Friends/family	0.0%	0.0%	6.7%
	Other	20.0%	0.0%	0.0%
Effect of social media on patient decisions	Effect	20.0%	50.0%	33.3%
	Little effect	30.0%	0.0%	33.3%
	Did not affect	50.0%	50.0%	33.3%
Effect of family opinion on patient decision	Effect	10.0%	50.0%	20.0%
	Little effect	40.0%	0.0%	13.3%
	Did not affect	50.0%	50.0%	66.7%
The extend to which patients’ expected outcomes affected their decision	Not important	10.0%	0.0%	0.0%
	Slightly important	0.0%	0.0%	6.7%
	Very important	50.0%*	0.0%	26.7%
	It was the main factor	40.0%	100.0%	66.7%
Patients’ major concerns about doing the surgery	Possible pain	30.0%	100.0%	20.0%
	Outcome	10.0%	0.0%	40.0%
	Nothing	40.0%	0.0%	33.3%
	Others	20.0%	0.0%	6.7%
Patients’ emotional expression prior surgery	Very anxious	0.0%	100.0%	13.3%
	Slightly anxious	0.0%	0.0%	13.3%
	Natural	50.0%	0.0%	60.0%*
	Excited	10.0%	0.0%	6.7%
	N\A	40.0%	0.0%	6.7%
Communication with the assigned Healthcare Team	Excellent	30.0%	100.0%	60.0%
	Good	30.0%	0.0%	13.3%
	Average	40.0%	0.0%	20.0%
	Poor	0.0%	0.0%	6.7%
Awareness of non-surgical alternatives to orthognathic surgery	Yes	80.0%	100.0%*	46.7%
	No	20.0%	0.0%	53.3%
If yes, where the options were considered	Yes	80.0%*	50.0%	33.3%
	No	0.0%	50.0%	13.3%
	N\A	20.0%	0.0%	53.3%
Accuracy level of the information given to patients regarding their surgery	Very accurate	40.0%	100.0%	46.7%
	Somewhat	50.0%	0.0%	40.0%
	Not accurate	10.0%	0.0%	13.3%
The influence of cultural beliefs and norms on patients’ surgery decision	Not at all	80.0%*	50.0%	60.0%
	Little	20.0%	50.0%	13.3%
	A lot	0.0%	0.0%	20.0%
	It was the main factor	0.0%	0.0%	6.7%
Any experience social stigma or negative reactions from others regarding your decision to undergo surgery	Yes	20.0%	0.0%	33.3%
	No	80.0%	100.0%	66.7%
Whether patients recommend orthognathic surgery to others after they received the treatment	Yes	20.0%	100.0%*	53.3%
	Maybe	80.0%	0.0%	46.7%

*P-value < 0.05.

Doctors were the primary source of information across all education levels, particularly for those with a university education (53.3%) and diploma holders (50.0%). Internet usage was highest among diploma holders (50.0%) and university-educated patients (33.3%), while high school graduates were less likely to use the Internet as a source of information (20.0%). The impact of social media was most significant among diploma holders (50.0%), followed by university-educated participants (33.3%) and high school graduates (20.0%).

Expected outcomes were the main factor that influenced the decision for 100.0% of diploma holders, compared to 66.7% of university-educated participants and 40.0% of high school graduates. Concerns about possible pain were significantly higher among diploma holders (100.0%) compared to participants with a university education (20.0%) and high school graduates (30.0%).

Emotional states before surgery varied, with all diploma holders reporting high levels of anxiety (100.0%), while high school graduates and university-educated participants were more likely to feel no anxiety (50.0% and 60.0%, respectively). Communication with the healthcare team was rated as excellent by all diploma holders, compared to 60.0% of university-educated participants and 30.0% of high school graduates.

Awareness of non-surgical alternatives was significantly higher among diploma holders (100.0%) and high school graduates (80.0%) compared to university-educated participants (46.7%). Information accuracy was deemed very accurate by 100.0% of diploma holders compared to 46.7% of university-educated patients and 40.0% of high school graduates. This was a significant difference. Cultural beliefs and norms had no influence on the decisions of 80.0% of high school graduates, compared to 60.0% of university-educated participants and 50.0% of diploma holders.

Social stigma or negative reactions were experienced by 33.3% of university-educated participants, compared to 20.0% of high school graduates and no diploma holders. After receiving treatment, 100.0% of diploma holders stated that they would recommend orthognathic surgery to others compared to 53.3% of university-educated participants and 20.0% of high school graduates, which was a significant difference (*p* < 0.05). Table 4 highlighted the statistical relationship among level of education and several variables.

### Impact of deformity type on orthognathic surgery decisions

The frequency distribution of various variables according to the type of deformity can be seen in Table 5. The impact of expected outcomes on decision-making varied across the deformity types, with 80.0% of participants with Class I deformities citing it as the main factor, compared to 66.7% of those with Class II and 50.0% of those with Class III deformities.

**Table 5.** Analysis of decision-making factors in relation to type of deformity.

Orthognathic Decision		Type of Deformity		
		Class I	Class II	Class III
The extend to which patients’ expected outcomes affected their decision	Not important	0.0%	0.0%	6.3%
	Slightly important	0.0%	16.7%	0.0%
	Very important	20.0%	16.7%	43.8%
	It was the main factor	80.0%	66.7%	50.0%
Patients’ major concerns about doing the surgery.	Possible pain	0.0%	50.0%	31.3%
	Outcome	20.0%	16.7%	31.3%
	Nothing	80.0%	33.3%	18.8%
	Others	0.0%	0.0%	18.8%
Awareness of non-surgical alternatives to orthognathic surgery	Yes	40.0%	66.7%	68.8%
	No	60.0%	33.3%	31.3%
Whether patients recommend orthognathic surgery to others after they received the treatment	Yes	20.0%	50.0%	50.0%
	Maybe	80.0%	50.0%	50.0%

Regarding major surgery concerns, the most prevalent concern among participants with Class II deformities was possible pain (50.0%), compared to 31.3% of those with Class III deformities. Interestingly, 80.0% of participants with Class I deformities reported no major concerns, compared to 33.3% of those with Class II and 18.8% of those with Class III deformities. Additionally, 18.8% of participants with Class III deformities had concerns categorised as ‘other’, while no such concerns were raised by those with Class I or Class II deformities.

When asked whether they would recommend orthognathic surgery to others after receiving treatment, 50.0% of participants with Class II and Class III deformities said they would, compared to only 20.0% of those with Class I deformities. The remaining patients across all deformity categories were uncertain, with 80.0% of those with Class I deformities and 50.0% of those with Class II and Class III deformities responding with ‘maybe’.

## Discussion

Orthognathic surgery has become increasingly relevant due to its potential to significantly enhance both the functional and aesthetic aspects of patients’ lives [9]. This study presents one of the few investigations focused on understanding the decision-making factors for orthognathic surgery in the Ha’il region, Saudi Arabia, filling a gap in the literature on patient motivations and influences in this specific cultural and healthcare context.

The results reveal that aesthetic concerns serve as a predominant motivator, with nearly half of the patients prioritizing improvements in appearance. This aligns with global findings, which suggest that facial aesthetics strongly influence the decision to undergo orthognathic surgery [10,11]. However, in contrast to findings from more economically developed regions, such as the United States or Europe, functional concerns played a relatively minor role in this population [12–16]. This difference may be attributed to varying levels of public awareness regarding the broader health benefits of orthognathic surgery, as well as the importance placed on aesthetics in Saudi culture [17,18].

The role of healthcare professionals emerged as a significant influence on patient decision-making, with the majority of patients receiving their information from doctors. This underscores the pivotal role healthcare teams play in guiding patients through the decision-making process. Moreover, the data highlights the need for improved patient education, as less than half of the participants reported receiving comprehensive information about the risks and benefits of the surgery [19]. This finding suggests a gap in patient-physician communication that could be addressed through targeted educational interventions, further supported by the goals of Saudi Vision 2030.

Interestingly, social stigma related to undergoing orthognathic surgery was a notable concern for a quarter of the participants [20]. This reveals an underlying social pressure that may influence a patient’s willingness to proceed with surgery, despite medical or aesthetic indications. Addressing this stigma through public health campaigns and normalizing the benefits of orthognathic surgery could potentially reduce this barrier [21,22].

While cultural beliefs were not a major factor for most patients, the data indicate that for a small subset, cultural and familial influences played a role. This finding suggests that, in some cases, external social factors, rather than personal desire or medical need, drive the decision-making process. This observation aligns with previous studies, which emphasize the varying impact of cultural norms across different populations [2,23,24].

The study’s primary limitation lies in its small sample size, which may affect the generalizability of the findings. Despite this, the insights gained provide a valuable foundation for future research on orthognathic surgery in Saudi Arabia and the broader Middle Eastern context. Further studies involving larger and more diverse populations are encouraged to deepen our understanding of the factors influencing patients’ decisions to undergo this transformative procedure.

## Conclusion

This study evaluated the prevalence and distribution of orthognathic surgeries performed between 2019–2023 in the Ha’il Region of Saudia Arabia. Age, gender, and educational levels impacted participants’ decision-making processes for orthognathic interventions, particularly among participants with Class III skeletal malocclusions. The current study highlighted the significance online sources and support from family and friends in decision-making. Additionally, dental professionals were found to significantly contribute to the decision-making processes of participants. Future advancements in healthcare could look to improve the processes and categories related to patient care to facilitate collaborative decision-making for patients needing orthognathic treatment.

## Data Availability

The datasets created and/or analysed for the current study are not publicly accessible because ethics approval was given on the grounds that only the researchers involved in the study would have access to the identified data, but they are available from the corresponding author upon justifiable request.
